# The mediation of systemic inflammation on insulin resistance and poor prognosis in non-diabetic ischemic stroke patients treated with intravenous thrombolysis

**DOI:** 10.3389/fneur.2025.1580862

**Published:** 2025-07-08

**Authors:** Yanli Sun, Wei Deng, Heng Wang, Mingwei Chen

**Affiliations:** ^1^Department of Endocrinology, The First Affiliated Hospital of Anhui Medical University, Hefei, Anhui, China; ^2^Department of General Practice, Xiangyang No.1 People's Hospital, Hubei University of Medicine, Xiangyang, Hubei, China; ^3^Department of Neurology, Xiangyang No.1 People's Hospital, Hubei University of Medicine, Xiangyang, Hubei, China; ^4^Center for Big Data and Population Health of IHM, The First Affiliated Hospital of Anhui Medical University, Hefei, Anhui, China

**Keywords:** triglyceride-glucose index, inflammatory markers, acute ischemic stroke, intravenous thrombolysis, insulin resistance

## Abstract

**Background and purpose:**

Insulin resistance (IR) has been linked to poor stroke prognosis even in non-diabetic patients, but the underlying mechanisms remain unclear. This study aims to explore whether the association between IR and poor prognosis in non-diabetic patients with acute ischemic stroke (AIS) treated with intravenous recombinant tissue-type plasminogen activator (IV-rtPA) is mediated by systemic inflammation.

**Methods:**

In this retrospective study, 841 consecutive patients with AIS but without a history of diabetes treated with IV-rtPA were included. IR was evaluated by means of the triglyceride-glucose index (TyG). Inflammatory markers, including the neutrophil-to-lymphocyte ratio (NLR), platelet-to-lymphocyte ratio (PLR), systemic immune-inflammation index (SII), and inflammation prognostic index (IPI), were calculated based on blood parameters obtained within 24 h of admission. The primary outcome was poor prognosis at 90 days [modified Rankin Scale (mRS) score ≥3]. Multivariable logistic regression analysis was performed to explore the associations among TyG, inflammatory markers, and the poor prognosis. A mediation analysis was performed to examine the relationship between IR and the study outcome mediated by systemic inflammation.

**Results:**

In total, 107 (12.72%) had poor prognosis. After adjusting for confounders (Model 3), multivariable logistic regression analysis revealed that both TyG and NLR were significantly associated with poor prognosis [odds ratio (OR), 2.212 (95% CI, 1.564–5.617), *P* < 0.001; 1.059 (95% CI, 0.904–1.241), *P* = 0.004; respectively]. Both indicators exhibited strong predictive value for poor prognosis, with areas under the curve (AUCs) of 0.823 and 0.730, respectively. Moreover, NLR and IPI were found to partially mediate the relationship between TyG and poor prognosis, with mediation proportions of 16.5 and 13.8%, respectively. After propensity score matching (PSM), the mediating effects of inflammatory markers became more pronounced.

**Conclusion:**

Our study found that insulin resistance was associated with poor prognosis in non-diabetic patients treated with IV-rtPA, and this association was partially mediated by NLR and IPI to a modest extent. These findings offer new insights into the clinical management of non-diabetic AIS patients after IV.

## Introduction

Stroke is a leading cause of death and disability in China, with ischemic stroke accounting for ~70% of all cases (1). Intravenous thrombolysis (IVT) is the primary treatment for acute ischemic stroke (AIS) within 4.5 h of onset (2), yet up to three-quarters of patients still experience poor outcomes (3), highlighting the urgent need to identify novel prognostic markers and pathophysiological mechanisms. Insulin resistance (IR), characterized by reduced tissue sensitivity to insulin and impaired glucose utilization, is commonly assessed using the triglyceride-glucose index (TyG) as a reliable indicator (4). Research shows that IR is the primary mechanism underlying the development of type 2 diabetes mellitus (T2DM) and also contributes to the onset of cerebrovascular diseases in non-T2DM patients (5, 6). Additionally, IR has been associated with recurrent strokes and poor functional prognosis in AIS (7, 8). However, there is limited research on the post-thrombolysis outcomes in non-diabetic ischemic stroke patients, and the underlying mechanisms linking IR to stroke outcomes remain unclear.

The comprehensive anabolic effects of insulin throughout the body, in addition to the control of glycemia, include ensuring lipid homeostasis and anti-inflammatory modulation (9). Recent studies suggest that systemic inflammation may serve as a key link between IR and cerebrovascular outcomes, as impaired insulin signaling can promote chronic low-grade inflammation (10, 11). Neuroinflammation is increasingly recognized as a critical driver of ischemic injury and post-stroke complications (12, 13). In this context, inflammatory markers such as the neutrophil-to-lymphocyte ratio (NLR) and platelet-to-lymphocyte ratio (PLR) have shown independent prognostic value in AIS patients (14, 15). Moreover, composite indices like the systemic immune-inflammation index (SII) and inflammation prognostic index (IPI), derived from peripheral blood counts may offer enhanced predictive performance for short-term stroke outcomes (16). These findings raise the possibility that systemic inflammation mediates the adverse effects of IR on AIS outcomes. However, the specific role of these inflammatory markers in mediating the association between IR and post-thrombolysis outcomes in non-diabetic patients remains unclear and warrants further investigation.

Therefore, this study aims to investigate the association between IR, inflammatory markers, and poor prognosis in non-diabetic patients with AIS treated with IV-rtPA. It further explores whether systemic inflammation mediates the relationship between IR and clinical outcomes.

## Methods

### Study design and participants

In this retrospective case-control study, data were collected from consecutive 1,181 patients with AIS treated with IV-rtPA at the Department of Neurology, Xiangyang No.1 People's Hospital, Hubei University of Medicine from January 2019 to December 2023. The Xiangyang No.1 People's Hospital Human Research Ethics Committee has approved the study protocol (Approval No. XYYYE20250018). The inclusion criteria were as follows: (1) age ≥18 years; (2) received IV-rtPA within 4.5 h of symptom onset at a standard dose of 0.9 mg/kg (maximum 90 mg), with 10% given as an initial bolus over 1 min and the remaining 90% infused continuously over 60 min; (3) pre-stroke modified Rankin Scale (mRS) ≤ 2; and (4) no history of diabetes. The exclusion criteria were: (1) patients who underwent bridging therapy; (2) those with malignant tumors, autoimmune diseases, or hematologic disorders; (3) patients with severe respiratory or urinary tract infections; (4) patients with severe hepatic or renal insufficiency; and (5) patients with incomplete clinical data.

### Data acquisition

Clinical data for the included patients were collected through electronic medical records. The data included age, gender, height, weight, National Institutes of Health Stroke Scale (NIHSS) score, mRS score, vascular risk factors (hypertension, diabetes, atrial fibrillation, coronary artery disease, previous stroke, smoking, and alcohol history), laboratory tests [hemoglobin, creatinine, fasting blood glucose, total cholesterol (TC), triglycerides (TG), low-density lipoprotein (LDL), high-density lipoprotein (HDL), etc.], and imaging tests (CT or MRI scans of the head). For outcome assessment, the 90-day mRS score was determined through standardized telephone interviews or outpatient follow-up conducted by trained neurologists who were blinded to baseline IR and inflammation status. A poor prognosis was defined as an mRS score of 3–6. According to the TOAST classification for AIS based on the ORG 10172 trial (17), the etiologies of AIS are categorized into large artery atherosclerosis (LAA), small artery occlusion (SAO), cardioembolism (CE), other determined etiology (SOE), and undetermined etiology (SUE).

### Measurement of TyG and inflammatory markers

Venous blood samples were collected within 24 h of hospital admission. If multiple blood tests were performed during this period, the results from the first test were used for analysis. The following laboratory parameters were recorded: fasting plasma glucose (FPG), triglycerides (TG), neutrophils (N), lymphocytes (L), platelets (PLT), albumin (ALB), and C-reactive protein (CRP). Based on these data, the following indices were calculated: triglyceride-glucose (TyG) index = ln [TG (mg/dl) × FPG (mg/dl)/2] (10); neutrophil-to-lymphocyte ratio (NLR = N/L); platelet-to-lymphocyte ratio (PLR = PLT/L); systemic immune-inflammation index (SII = PLT × N/L); and inflammation prognostic index (IPI = CRP × NLR/ALB).

### Statistical analysis

Continuous variables with a normal distribution are presented as mean ± SD, while those that do not follow a normal distribution are presented as median and interquartile range (IQR). Categorical variables are expressed as frequency and percentage. Differences between two groups were compared using Student's *t*-test, Mann–Whitney *U* test, or Chi-square test. Receiver operating characteristic (ROC) curve analysis was used to determine the cutoff values for each index in predicting poor prognosis, and the sensitivity and specificity of these values were calculated.

To mitigate multicollinearity among TyG and inflammatory markers, stepwise logistic regression based on the Akaike Information Criterion was employed to identify the optimal set of predictors. Logistic regression models were then used to estimate odds ratios (ORs) and 95% confidence intervals (CIs) for poor prognosis. Model 1 was unadjusted. Model 2 was adjusted for age and sex. Model 3 was further adjusted for age, sex, admission NIHSS score, smoking status, alcohol consumption, TOAST classification, and comorbidities. To address potential confounding and bias inherent in observational studies, propensity score matching (PSM) was conducted as a robustness analysis. A 2:1 nearest-neighbor matching algorithm with a caliper width of 0.2 was applied to balance key prognostic covariates between groups. Subsequently, subgroup analyses were performed to evaluate the consistency of the association between the TyG index and poor prognosis across various clinical strata after PSM.

**Figure 1:**
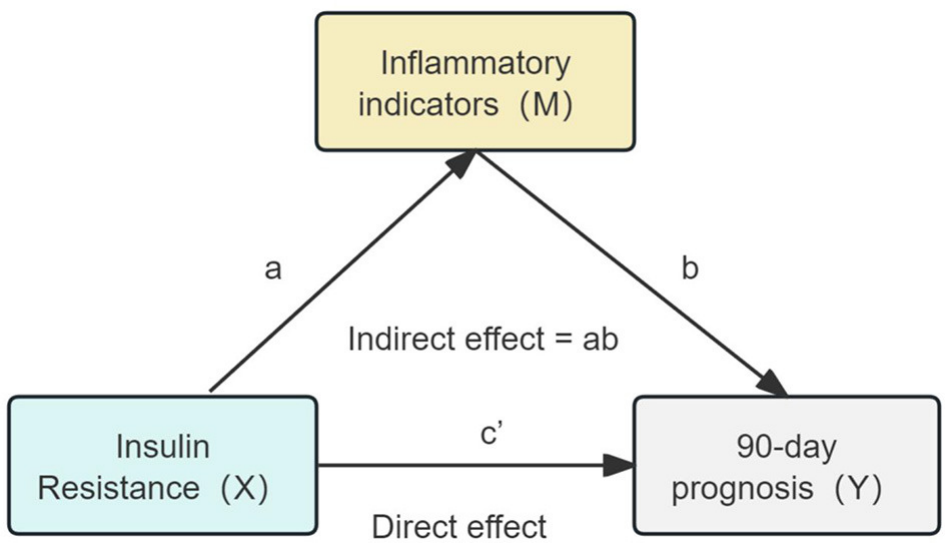
Hypothetical causal pathway model in non-diabetic ischemic stroke patients treated with intravenous thrombolysis. The total effect (c) is decomposed into the natural direct effect (c′) and the natural indirect effect (ab). TyG represents insulin resistance (X); inflammatory markers (M) include NLR, PLR, SIRI, SII, and IPI; the dependent variable (Y) represents poor prognosis defined as mRS ≥ 3.

Spearman correlation analysis was first conducted to examine the pairwise relationships between the TyG index and inflammatory markers (CRP, NLR, PLR, and IPI). Only markers demonstrating significant sequential associations were included in subsequent mediation models. To explore the mediating role of inflammatory markers in the association between IR and poor prognosis, causal mediation analysis was conducted within a counterfactual framework, based on Baron and Kenny's regression approach and bootstrap estimation. Specifically, the total effect of TyG on poor prognosis (c) was decomposed into the direct effect (c′) and the indirect effect (ab) through each inflammatory mediator (M). The proportion mediated was calculated as the ratio of the indirect effect to the total effect (ab/c). All models were adjusted for potential confounders including sex, age, admission NIHSS score, smoking status, alcohol consumption, TOAST classification, and comorbidities. Logistic regression was applied to binary outcomes, and indirect effects were estimated using a non-parametric bootstrap procedure with 5,000 iterations to obtain robust 95% confidence intervals. The proposed causal pathway is shown in Figure 1.

We used SPSS Statistics version 25.0 (SPSS Inc., Chicago, IL, USA) for data analysis and R version 4.1.0 (R Foundation for Statistical Computing, Vienna, Austria) for plotting and statistical analysis. A two-tailed *P*-value < 0.05 was considered statistically significant.

## Results

This study screened 853 non-diabetic ischemic stroke patients who received IVT. Due to exclusion criteria including bridging therapy after IVT (*n* = 6), malignancy (*n* = 1), severe respiratory or urinary tract infections (*n* = 3), and incomplete clinical data (*n* = 2), a total of 12 patients were excluded. Ultimately, 841 participants were included in the final analysis. The patient selection flowchart is shown in Supplementary Figure S1.

### Characteristics of participants

**Figure 2:**
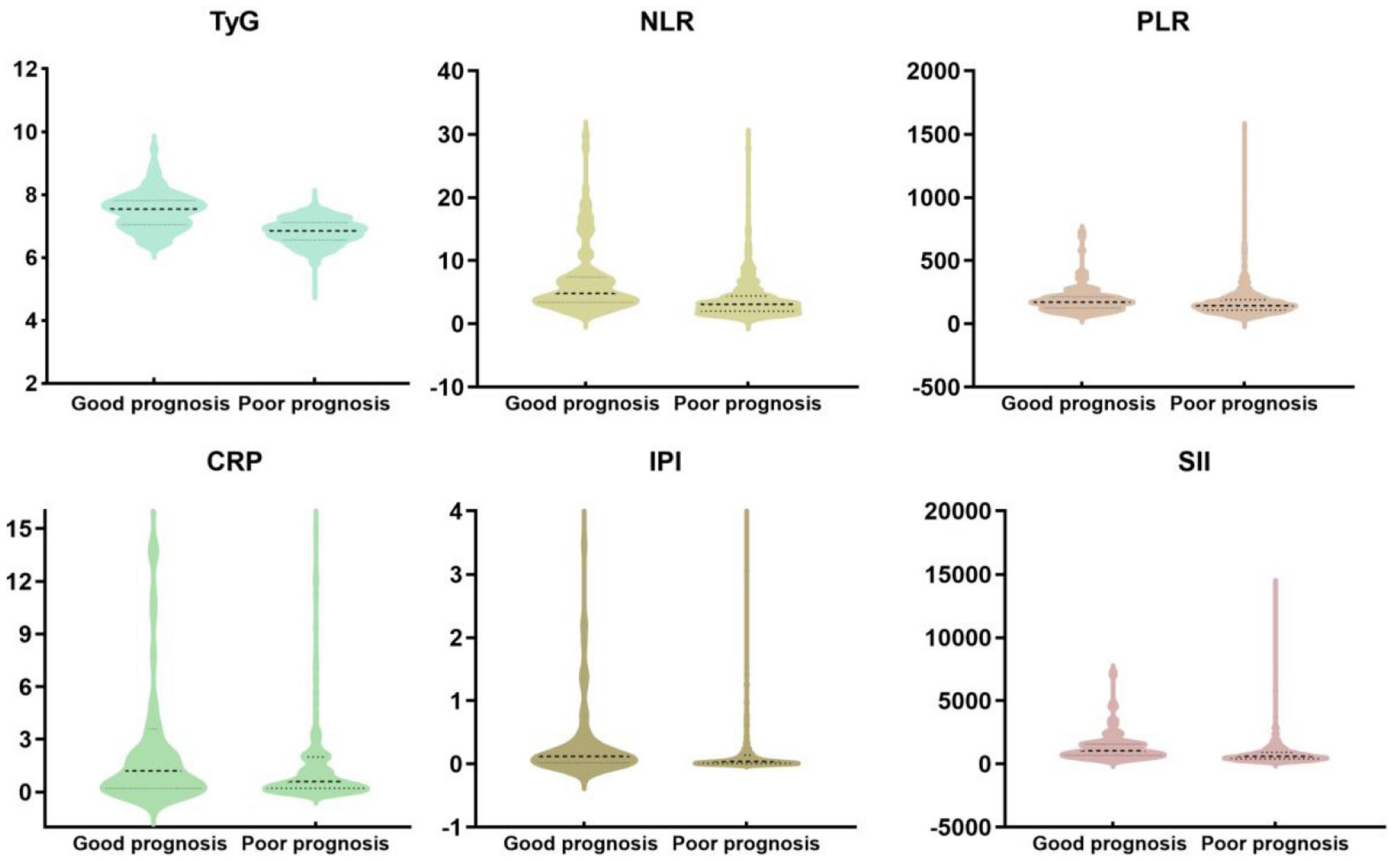
The violin plot in distribution of the inflammatory markers (TyG, NLR, PLR, SIRI, SII, and IPI) between the two groups.

Table 1 presents the baseline characteristics of the study participants. Compared to the good prognosis group, the poor prognosis group had higher age [66 (58, 73) vs. 70 (62, 78), *P* < 0.001], BMI [23.53 (21.1, 25.39) vs. 25.48 (23.74, 27.24), *P* < 0.001], hypertension (65.4% vs. 77.6%, *P* = 0.012), atrial fibrillation (6 vs. 21.5%, *P* < 0.001). There were no statistically significant differences between the two groups in terms of DNT time (*P* = 0.952). Figure 2 shows violin plots comparing the TyG, NLR, PLR, SIRI, SII, and IPI levels between the two groups. The levels of TyG, NLR, PLR, SIRI, SII, and IPI were lower in the good prognosis group compared to the poor prognosis group (*P* < 0.05). After propensity score matching, baseline characteristics were generally well-balanced between groups. Detailed results are presented in Supplementary Table S1.

**Table 1:** Demographics and clinical characteristics of AIS patients with different prognosis groups [*n* (%), mean ± SD, median (IQR)].

**Characteristics**	**Totality (*n* = 841)**	**Good prognosis group (*n* = 734)**	**Poor prognosis group (*n* = 107)**	***P* value**
**Demographic data**				
Sex men	493 (58.6%)	424 (57.8%)	69 (64.5%)	0.187
Age (year)	67 (58, 74)	66 (58–73)	70 (62–78)	<0.001
**Stroke risk factors**				
Smoking	288 (34.2%)	243 (33.1%)	45 (42.1%)	0.068
Drinking history	230 (27.3%)	195 (26.6%)	35 (32.7%)	0.387
Hypertension	563 (66.9%)	480 (65.4%)	83 (77.6%)	0.012
CHD	99 (11.8%)	81 (11%)	18 (18.8%)	0.083
Atrial fibrillation	67 (8%)	44 (6%)	23 (21.5%)	<0.001
Hyperlipemia	192 (22.8%)	169 (23%)	23 (21.5%)	0.725
History of stroke	32 (3.8%)	26 (3.5%)	6 (5.6%)	0.297
**Clinical data**				
BMI (kg/m^2^)	23.73 (21.48–25.65)	23.53 (21.1–25.39)	25.48 (23.74–27.24)	<0.001
SBP (mmHg)	143.84 ± 20.05	147.79 ± 24.0.96	143.27 ± 19.18	0.029
DBP (mmHg)	82 (74–92)	81 (75–91)	84 (71–98)	0.547
**TOAST classification**				
LAA	200 (23.8%)	160 (21.8%)	40 (37.4%)	<0.001
SAO	561 (66.7%)	516 (70.3%)	45 (42.1%)	
CE	62 (7.4%)	41 (5.6%)	21 (19.6%)	
SOE + SUE	18 (2.1%)	17 (2.3%)	1 (0.9%)	
DNT (min)	38 (35, 42)	38 (32, 45)	38 (35, 42)	0.952
Admission NIHSS score	3 (2–5)	3 (2–5)	9 (4–13)	<0.001
**Laboratory data**				
LDL-C (mmol/L)	2.46 (1.98–2.98)	2.47 (1.98–2.98)	2.33 (1.98–3.08)	0.296
HDL (mmol/L)	1.15 (0.98–1.34)	1.15 (0.97–1.33)	1.16 (1–1.37)	0.58
CRP (mg/L)	0.64 (0.21, 2)	0.6 (0.21, 2)	1.21 (0.21, 3.6)	0.006
TyG	6.89 (6.59, 7.2)	6.85 (6.56, 7.11)	7.54 (7.04, 7.81)	<0.001
NLR	3.2 (2.15–4.88)	3.1 (2.01–4.43)	4.85 (3.4–7.41)	<0.001
PLR	146.72 (110.41–195.37)	144.75 (108.65–191.26)	172.54 (125.82–213.82)	0.001
SII	639.89 (432.3–1,009.69)	592.57 (399.12–925.68)	1,036.33 (680–1,575.41)	<0.001
IPI	0.04 (0.01–0.16)	0.04 (0.01–0.14)	0.12 (0.01–0.74)	<0.001

BMI, body mass index; SBP, systolic blood pressure; DBP, diastolic blood pressure; CHD, coronary heart disease; LAA, large artery atherosclerosis; SAO, small artery occlusion; CE, cardioembolism; SOE, stroke of other determined etiology; SUE, stroke of undetermined etiology; DNT, door-to-needle time; LDL-C, low-density lipoprotein cholesterol; HDL-C, high-density lipoprotein cholesterol.

### Prognostic value of TyG, inflammatory markers in non-diabetic ischemic stroke patients treated with IVT

**Figure 3:**
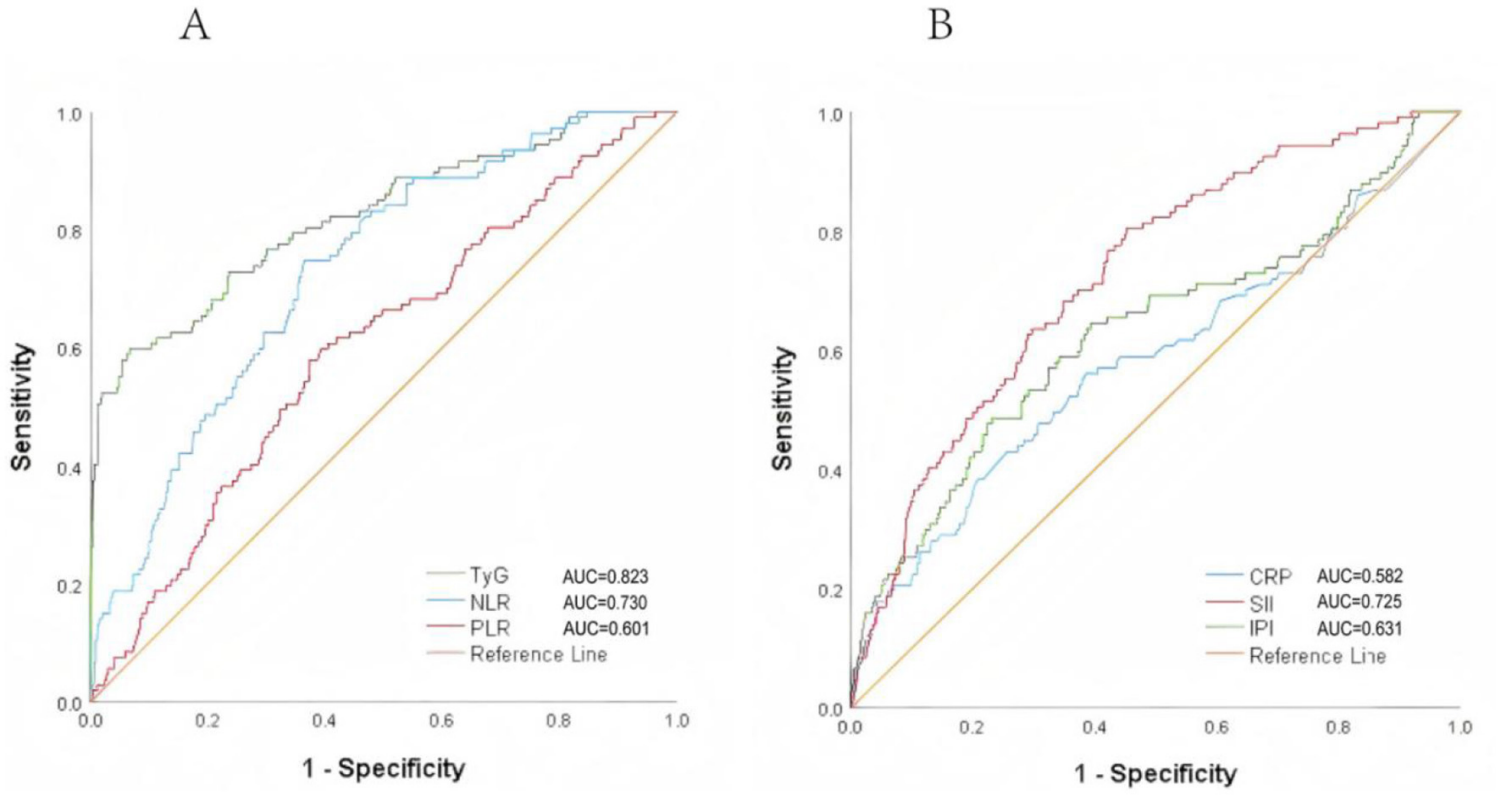
ROC curves of TyG, NLR, PLR, SIRI, SII, and IPI for predicting poor prognosis in non-diabetic ischemic stroke patients treated with intravenous thrombolysis. **(A)** The AUC of TyG was 0.823, with a sensitivity of 0.589 and a specificity of 0.933. The AUC of NLR was 0.730, with a sensitivity of 0.748 and a specificity of 0.636. **(B)** The AUC of SII was 0.725, with a sensitivity of 0.804 and a specificity of 0.649.

Figure 3 shows the ROC chart the lower area of TyG index predicting poor prognosis was [AUC 0.823 (95% CI 0.775–0.871)], the Yoden index was 0.531, and the cutoff point was 7.378, *P* < 0.001 (sensitivity = 0.589, specificity = 0.933). The prognostic value of NLR in predicting poor prognosis [AUC 0.73 (95% CI 0.682 to 0.777)], Jordon index 0.384, cut-off point 3.563, *P* < 0.001 (sensitivity = 0.748; specificity = 0.636); prognostic value of PLR in predicting poor prognosis [AUC 0.601 (95% CI 0.544–0.657)], Yoden index 0.206, cutoff point 161.943, *P* = 0.001 (sensitivity = 0.579; specificity = 0.61.3), the lower area of CRP predicting poor prognosis was [AUC 0.582 (95% CI 0.517–0.646)], Yoden index was 0.176, cutoff point was 1.035, *P* = 0.006 (sensitivity = 0.385, specificity = 0.824). The prognostic value of SII in predicting poor prognosis [AUC 0.725 (95% CI 0.676–0.774)], the Yodon index was 0.353, the cutoff point was 645.602, *P* < 0.001 (sensitivity = 0.804; specificity = 0.649); the prognostic value of IPI for predicting poor prognosis [AUC 0.631 (95% CI 0.5688–0.694)], Yoden index 0.254, cut-off point 0.152, *P* < 0.001 (sensitivity = 0.486; specificity = 0.768).

### Univariate and multivariate logistic analysis of TyG, NLR, PLR, IPI, CRP, and poor prognosis in non-diabetic ischemic stroke patients treated with IVT

In the univariate regression analysis, the continuous variables TyG, NLR, PLR, and IPI at admission were significantly associated with poor prognosis at 3 months (*P* < 0.05). In the binary logistic regression analysis, TyG, and NLR were significantly associated with the outcome in both continuous and categorical models (Model 1, Model 2, and Model 3; *P* < 0.05), with the effect of TyG being the most significant (*P* < 0.001). The effects of PLR and IPI were weaker, showing significance in the continuous variables model (*P* < 0.05), but not in the categorical variables model. CRP did not show a significant effect in any of the models (Table 2).

**Table 2:** Univariate and multivariate logistic regression analyses of TyG index and inflammatory markers for poor prognosis in non-diabetic patients treated with IV.

**Exposure**	**Model 1**		**Model 2**		**Model 3**	
	**OR (95% CI)**	***P*** **-value**	**OR (95% CI)**	***P*** **-value**	**OR (95% CI)**	***P*** **-value**
**TyG**						
Continuous	2.101 (1.731–5.616)	<0.001	2.611 (1.419–5.921)	<0.001	2.212 (1.564–5.617)	<0.001
**Categorical**						
TyG < 7.378	–		–		–	
TyG > 7.378	2.283 (1.898–3.672)	<0.001	2.514 (1.446–3.269)	<0.001	2.638 (1.464–4.709)	<0.001
**NLR**						
Continuous	1.202 (1.1–1.312)	<0.001	1.207 (1.102–1.322)	<0.001	1.059 (0.904–1.241)	0.004
**Categorical**						
NLR < 3.563	–		–		–	
NLR > 3.563	2.439 (1.115–5.333)	0.026	2.26 (1.028–4.968)	0.003	2.035 (0.901–4.596)	0.008
**PLR**						
Continuous	0.995 (0.991–0.999)	0.015	0.996 (0.992–1.00)	0.044	0.993 (0.988–0.999)	0.014
**Categorical**						
PLR < 161.94	–		–		–	
PLR > 161.94	0.877 (0.497–1.549)	0.652	1.073 (1.024–1.125)	0.531	0.812 (0.4350–1.515)	0.512
**CRP**						
Continuous	0.956 (0.912–1.002)	0.062	0.958 (0.913–1.005)	0.077	0.955 (0.908–1.004)	0.07
**Categorical**						
CRP < 1.035	–		–		–	
CRP > 1.035	1.324 (0.062–2.911)	0.485	0.831 (0.539–2.689)	0.65	1.129 (0.474–2.689)	0.784
**IPI**						
Continuous	1.364 (1.0.56–1.762)	0.018	1.073 (1.024–1.125)	0.003	1.327 (1.016–1.733)	0.038
**Categorical**						
IPI < 0.254	–		–		–	
IPI > 0.254	1.08 (0.453–2.578)	0.862	1.063 (0.44–2.568)	0.892	0.98 (0.371–2.591)	0.968

Model 1: no adjustment for covariates.

Model 2: adjusted for sex and age.

Model 3: adjusted for sex, age, admission NIHSS score, smoking status, alcohol consumption, TOAST classification, and comorbidities. High TyG, NLR and SII (P < 0.05) were independent predictors of poor prognosis, according to multivariate logistic regression analysis.

OR, odds ratio; CI, confidence interval.

The results after regression analysis following propensity score matching were generally consistent with the primary outcome after multifactor adjustments. Details are available in Supplementary Table S2. The association between elevated TyG index and poor 90-day outcomes remained robust across subgroups stratified by age, hypertension, CHD, SBP, BMI, and admission NIHSS score after PSM, with no significant effect modification detected (Supplementary Figure S2).

### Mediation analysis

TyG was significantly associated with results in both continuous and categorical variable models (*P* < 0.05), suggesting that IR was independently associated with poor prognosis. In addition, Spearman correlation analysis showed that TyG index was significantly correlated with CRP, NLR, SII, and IPI (*P* < 0.05), among which SII had the strongest correlation (*P* = 0.003), but was not significantly correlated with PLR (*P* = 0.565; Table 3).

**Table 3:** Spearman correlation coefficients between TyG index and inflammatory markers.

	**TyG**	**CRP**	**NLR**	**PLR**	**IPI**
TyG	1	0.076^*^	0.078^*^	0.02	0.074^*^
CRP	0.076^*^	1	0.103^**^	0.019	0.947^**^
NLR	0.078^*^	0.103^**^	1	0.674^**^	0.367^**^
PLR	0.02	0.019	0.674^**^	1	0.200^**^
IPI	0.074^*^	0.947^**^	0.367^**^	0.200^**^	1

* *P* < 0.05;

** *P* < 0.01.

The mediation analysis results indicate that systemic inflammation plays a significant partial mediating role in the association between TyG and poor prognosis in non-diabetic ischemic stroke patients after IVT. In the adjusted model 3, inflammation markers as mediators significantly influenced the relationship between TyG and poor prognosis. Specifically, the results show that ~16.5% of the total effect of TyG on 90-day prognosis was mediated by NLR, and about 13.8% was mediated by IPI (both *P* < 0.001; Table 4). After propensity score matching, the mediating effects of inflammatory markers became more pronounced, with NLR and IPI mediating 19.2 and 15.9% of the total effect of TyG on 90-day poor prognosis, respectively (both *P* < 0.01, Supplementary Table S3).

**Table 4:** Mediation analysis of the effect of TyG on poor prognosis by inflammatory markers.

**Mediator**	**Total effect**		**Indirect effect**		**Direct effect**		**Proportion mediated**
	**Coefficient (95% CI)**	***P*** **-value**	**Coefficient (95% CI)**	***P*** **-value**	**Coefficient (95% CI)**	***P*** **-value**	
NLR	0.334 (0.225–0.460)	<0.001	0.055 (0.025–0.101)	0.001	0.279 (0.182–0.410)	<0.001	16.50%
IPI	0.333 (0.222–0.456)	<0.001	0.046 (0.018–0.089)	0.006	0.287 (0.186–0.416)	<0.001	13.80%

The total effect coefficient represents the overall impact of TyG on poor prognosis in Non-diabetic Patients treated with IV, encompassing both direct and indirect effects. The indirect effect quantifies the portion of this impact mediated through factors like NLR and IPI. The direct effect measures the impact of TyG on poor prognosis independent of the mediator. The proportion mediated indicates the fraction of the total effect explained by the mediator. TyG was considered as a continuous variable in the mediation analysis. The mediation analyses were adjusted for sex, age, admission NIHSS score, smoking, alcohol consumption, TOAST classification, and comorbidities.

## Discussion

In this retrospective study, we found that TyG, an alternative marker of IR, was strongly associated with poor prognosis in non-diabetic patients treated with IVT. Furthermore, TyG, NLR, and SII exhibited high predictive value for short-term poor outcomes, with AUCs of 0.823, 0.730, and 0.725, respectively. In addition, mediation analysis showed that NLR and IPI partially mediated the association between the TyG index and poor prognosis (16.5 and 13.8%, respectively), suggesting that some unrecognized mechanisms may also contribute to the outcomes of non-diabetic ischemic stroke patients treated with IVT.

As is well-known, IR is not only a key factor in metabolic disorders but also closely associated with various pathological processes such as lipid metabolism abnormalities, endothelial dysfunction, proliferation of vascular smooth muscle and mesenchymal cells, inflammatory responses, hypercoagulability, atherosclerosis, and thrombosis (6, 18). These factors are recognized as risk factors for poor prognosis in ischemic stroke. Previous studies have reported that IR is associated with different clinical outcomes in non-diabetic ischemic stroke patients (19, 20). However, research on the relationship between IR, inflammatory markers, and short-term outcomes in non-diabetic ischemic stroke patients treated with IVT remains relatively limited. Our study suggests that IR, represented by TyG, as well as NLR, is closely related to poor prognosis in these patients. A cohort study based on the third China National Stroke Registry (CNSR-III) found that higher HOMA-IR quartiles were associated with an increased risk of stroke recurrence, ischemic stroke, and composite vascular events within 1 year in non-diabetic ischemic stroke patients, particularly in the large artery atherosclerosis subtype (21). Another study demonstrated that a higher TyG index was associated with a poor 90-day outcomes in acute large vessel occlusion stroke patients after receiving endovascular therapy (22). Additionally, research analyzing data from the MIMIC-IV database found that TyG was significantly associated with hospital and ICU mortality in critically ill ischemic stroke patients (23). Our study also showed that the TyG index has high predictive value for poor prognosis (AUC = 0.823), which is consistent with previous findings (21–23). Therefore, TyG could potentially serve as a powerful tool for identifying high-risk stroke patients, providing support for more precise monitoring or early intervention.

In recent years, neuroinflammation has attracted increasing attention, as it plays a critical role in the pathogenesis and progression of AIS. In our study, we found that inflammatory markers were not only associated with poor prognosis in non-diabetic AIS patients after IVT, but also significantly correlated with insulin IR, suggesting a possible mediating role. IR is known to trigger a chronic low-grade inflammatory state by activating proinflammatory signaling pathways such as NF-κB, leading to increased release of cytokines like IL-6 and TNF-α. These inflammatory cytokines can disrupt endothelial function, increase blood-brain barrier permeability, and promote thrombogenesis—all of which exacerbate ischemic brain injury and impair neurological recovery (9, 10). During the acute phase of stroke, damage to brain tissue further releases chemokines and cytokines, resulting in the recruitment of inflammatory cells. Neutrophils rapidly accumulate in the infarct core and penumbra (24), releasing free radicals and proteolytic enzymes that aggravate tissue injury (25, 26). Conversely, lymphocytes are considered neuroprotective, and their relative depletion reflects an imbalance in immune homeostasis (27). Platelet activation and dysfunction are also implicated in post-stroke inflammation and microvascular thrombosis (28).

Composite inflammatory indices such as NLR, PLR, SII, and IPI integrate multiple immune pathways and can be easily derived from routine blood tests. Previous studies have demonstrated their predictive value for AIS outcomes (14–16, 29), and our study confirmed that NLR and SII had relatively high predictive value for poor functional outcome (AUCs of 0.73 and 0.725, respectively). These markers may reflect the degree of systemic inflammation driven both by stroke pathology and pre-existing metabolic disturbances such as IR. Elevated NLR and PLR may be associated with symptomatic internal carotid artery stenosis (30), while increased PLR levels are related to post-stroke depression (31). In AIS patients treated with IVT, NLR and PLR are associated with early neurological deterioration (END), and NLR is linked to early neurological improvement (ENI). Both NLR and PLR may have predictive capabilities for END following thrombolysis (29). Further studies have found that SII and IPI are independently associated with short-term outcomes in AIS patients, and they perform well in predicting 90-day outcomes (16). High SII indicates a thrombotic and immune dysregulation state, both of which are associated with severe adverse outcomes (32). IPI is calculated based on CRP, NLR, and albumin. Albumin, with its antioxidant and anti-inflammatory properties, is neuroprotective (33–35), while CRP is a commonly used inflammatory marker in clinical practice to reflect the degree of inflammation at the onset of infection and is associated with the severity, infarct size, and prognosis of AIS patients (36). However, in our study, CRP was not significantly associated with clinical outcomes, possibly due to patient selection and timing of measurement. Similarly, PLR and IPI showed weaker predictive power in our thrombolysis study, which may differ from findings in non-thrombolysis patients after IV due to clinical heterogeneity.

Our findings support the hypothesis that systemic inflammation mediates the relationship between insulin resistance (IR) and poor prognosis in non-diabetic AIS patients. IR promotes a chronic proinflammatory state that may exacerbate vascular injury and hinder neurological recovery. Notably, studies have shown that pretreatment with statins, which possess both anti-atherosclerotic and anti-inflammatory properties, is associated with improved recovery and reduced short-term mortality in AIS patients receiving thrombolysis (37). These findings suggest that targeting inflammation may help mitigate the detrimental effects of IR on stroke prognosis.

Our study has several limitations. First, it is a single-center, retrospective observational study. Patients without TyG index or inflammatory marker data were excluded, which may have introduced selection bias. Additionally, the observed mediating effects were modest and should be validated in multicenter, prospective studies. Second, although the TyG index is widely accepted as a surrogate marker for IR, it may not fully capture its complexity. Direct measurement methods, while more accurate, are invasive and less feasible in clinical practice. Third, we included five commonly used inflammatory markers (NLR, PLR, CRP, SII, and IPI); however, systemic inflammation involves complex pathways, and these markers may not fully reflect the overall inflammatory burden associated with IR. Fourth, as TyG and inflammatory markers were measured at admission or before thrombolysis, establishing a clear temporal sequence between exposure and mediation remains challenging. Future studies should consider serial measurements to better evaluate dynamic changes over time. Fifth, functional outcomes were assessed at 90 days, which may not fully reflect long-term prognosis. Longer follow-up is warranted to explore sustained effects of IR and inflammation on stroke recovery. Sixth, all participants were Chinese patients from a single center who received IVT, which may limit the generalizability of our findings to populations with different ethnic, regional, or genetic backgrounds. Future studies involving more diverse cohorts are needed to validate and extend our conclusions.

## Conclusion

To our knowledge, this is the first study to explore whether the association between IR and poor prognosis in non-diabetic patients after IVT is mediated by systemic inflammation. The TyG index and inflammatory markers are readily available clinical blood indicators, which were found to be significantly associated with poor prognosis, partly mediated by NLR and IPI. Additionally, we found that TyG, and NLR have high predictive value for poor functional outcome in thrombolysis patients. These findings provide new perspectives for the clinical management of non-diabetic ischemic stroke patients treated with IVT.

## Data Availability

The raw data supporting the conclusions of this article will be made available by the authors, without undue reservation.

## References

[1] Wang YJ Li ZX Gu HQ Zhai Y Jiang Y Zhao XQ. China stroke statistics 2019: a report from the National Center for Healthcare Quality Management in neurological diseases. Stroke Vasc Neurol. (2020) 5:211–39. 10.1136/svn-2020-000457 32826385 PMC7548521

[2] George BP Asemota AO Dorsey ER Haider AH Smart BJ Urrutia VC. United States trends in thrombolysis for older adults with acute ischemic stroke. Clin Neurol Neurosurg. (2015) 139:16–23. 10.1016/j.clineuro.2015.08.031 26363362

[3] Sandercock P Wardlaw JM Lindley RI Dennis M Cohen G Murray G. The benefits and harms of intravenous thrombolysis with recombinant tissue plasminogen activator within 6 h of acute ischaemic stroke (the third international stroke trial [IST-3]): a randomised controlled trial. Lancet. (2012) 379:2352–63. 10.1016/S0140-6736(12)60768-5 22632908 PMC3386495

[4] Du T Yuan G Zhang M Zhou X Sun X Yu X. Clinical usefulness of lipid ratios, visceral adiposity indicators, and the triglycerides and glucose index as risk markers of insulin resistance. Cardiovasc Diabetol. (2014) 13:146. 10.1186/PREACCEPT-1207347889137599 25326814 PMC4209231

[5] Hadwen J Kim W Dewar B Ramsay T Davis A Dowlatshahi D. Association between insulin resistance and post-ischaemic stroke outcome in patients without diabetes: protocol for a systematic review and meta-analysis. BMJ Open. (2021) 11:e044771. 10.1136/bmjopen-2020-044771 33771829 PMC8006852

[6] Jing J Pan Y Zhao X Zheng H Jia Q Mi D. Insulin resistance and prognosis of nondiabetic patients with ischemic stroke: the ACROSS-China Study (Abnormal Glucose Regulation in Patients With Acute Stroke Across China). Stroke. (2017) 48:887–93. 10.1161/STROKEAHA.116.015613 28235959

[7] Ago T Matsuo R Hata J Wakisaka Y Kuroda J Kitazono T. Fukuoka Stroke Registry I. Insulin resistance and clinical outcomes after acute ischemic stroke. Neurology. (2018) 90:e1470–7. 10.1212/WNL.0000000000005358 29602916

[8] Hardy OT Czech MP Corvera S. What causes the insulin resistance underlying obesity? Curr Opin Endocrinol Diabetes Obes. (2012) 19:81–7. 10.1097/MED.0b013e3283514e13 22327367 PMC4038351

[9] Szukiewicz D. Molecular mechanisms for the vicious cycle between insulin resistance and the inflammatory response in obesity. Int J Mol Sci. (2023) 24:9818. 10.3390/ijms24129818 37372966 PMC10298329

[10] Rehman K Akash MS. Mechanisms of inflammatory responses and development of insulin resistance: how are they interlinked? J Biomed Sci. (2016) 23:87. 10.1186/s12929-016-0303-y 27912756 PMC5135788

[11] Muniyappa R Chen H Montagnani M Sherman A Quon MJ. Endothelial dysfunction due to selective insulin resistance in vascular endothelium: insights from mechanistic modeling. Am J Physiol Endocrinol Metab. (2020) 319:E629–46. 10.1152/ajpendo.00247.2020 32776829 PMC7642854

[12] Parikh NS Merkler AE Iadecola C. Inflammation, autoimmunity, infection, and stroke: epidemiology and lessons from therapeutic intervention. Stroke. (2020) 51:711–8. 10.1161/STROKEAHA.119.024157 32078460 PMC7041866

[13] Dong X Gao J Zhang CY Hayworth C Frank M Wang Z. Neutrophil membrane-derived nanovesicles alleviate inflammation to protect mouse brain injury from ischemic stroke. ACS Nano. (2019) 13:1272–83. 10.1021/acsnano.8b06572 30673266 PMC6424134

[14] Zhu B Pan Y Jing J Meng X Zhao X Liu L. Neutrophil counts, neutrophil ratio, and new stroke in minor ischemic stroke or TIA. Neurology. (2018) 90:e1870–8. 10.1212/WNL.0000000000005554 29678934

[15] Lux D Alakbarzade V Bridge L Clark CN Clarke B Zhang L. The association of neutrophil-lymphocyte ratio and lymphocyte-monocyte ratio with 3-month clinical outcome after mechanical thrombectomy following stroke. J Neuroinflammation. (2020) 17:60. 10.1186/s12974-020-01739-y 32070366 PMC7026966

[16] Ma F Li L Xu L Wu J Zhang A Liao J. The relationship between systemic inflammation index, systemic immune-inflammatory index, and inflammatory prognostic index and 90-day outcomes in acute ischemic stroke patients treated with intravenous thrombolysis. J Neuroinflammation. (2023) 20:220. 10.1186/s12974-023-02890-y 37777768 PMC10543872

[17] Adams HP Jr Bendixen BH Kappelle LJ Biller J Love BB Gordon DL. Classification of subtype of acute ischemic stroke. Definitions for use in a multicenter clinical trial. TOAST. Trial of Org 10172 in Acute Stroke Treatment. Stroke. (1993) 24:35–41. 10.1161/01.STR.24.1.35 7678184

[18] Ormazabal V Nair S Elfeky O Aguayo C Salomon C Zuñiga FA. Association between insulin resistance and the development of cardiovascular disease. Cardiovasc Diabetol. (2018) 17:122. 10.1186/s12933-018-0762-4 30170598 PMC6119242

[19] Chang Y Kim CK Kim MK Seo WK Oh K. Insulin resistance is associated with poor functional outcome after acute ischemic stroke in non-diabetic patients. Sci Rep. (2021) 11:1229. 10.1038/s41598-020-80315-z 33441784 PMC7806587

[20] Jin A Wang S Li J Wang M Lin J Li H. Mediation of systemic inflammation on insulin resistance and prognosis of nondiabetic patients with ischemic stroke. Stroke. (2023) 54:759–69. 039542. 10.1161/STROKEAHA.122.039542 36722344

[21] Sun W Shen H Wu X He A Yao X Chen F. Influence of TyG index on large vascular occlusive stroke following endovascular treatment. CNS Neurosci Ther. (2024) 30:e70143. 10.1111/cns.70143 39648362 PMC11625684

[22] Cai W Xu J Wu X Chen Z Zeng L Song X. Association between triglyceride-glucose index and all-cause mortality in critically ill patients with ischemic stroke: analysis of the MIMIC-IV database. Cardiovasc Diabetol. (2023) 22:138. 10.1186/s12933-023-01864-x 37312120 PMC10262584

[23] Si S Li J Li Y Li W Chen X Yuan T. Causal effect of the triglyceride-glucose index and the joint exposure of higher glucose and triglyceride with extensive cardio-cerebrovascular metabolic outcomes in the UK biobank: a Mendelian Randomization Study. Front Cardiovasc Med. (2021) 7:583473. 10.3389/fcvm.2020.583473 33553250 PMC7863795

[24] Iadecola C Anrather J. The immunology of stroke: from mechanisms to translation. Nat Med. (2011) 17:796–808. 10.1038/nm.2399 21738161 PMC3137275

[25] Santos-Lima B Pietronigro EC Terrabuio E Zenaro E Constantin G. The role of neutrophils in the dysfunction of central nervous system barriers. Front Aging Neurosci. (2022) 14:965169. 10.3389/fnagi.2022.965169 36034148 PMC9404376

[26] Ceulemans AG Zgavc T Kooijman R Hachimi-Idrissi S Sarre S Michotte Y. The dual role of the neuroinflammatory response after ischemic stroke: modulatory effects of hypothermia. J Neuroinflammation. (2010) 7:74. 10.1186/1742-2094-7-74 21040547 PMC2988764

[27] Macrez R Ali C Toutirais O Le Mauff B Defer G Dirnagl U. Stroke and the immune system: from pathophysiology to new therapeutic strategies. Lancet Neurol. (2011) 10:471–80. 10.1016/S1474-4422(11)70066-7 21511199

[28] Xu XR Zhang D Oswald BE Carrim N Wang X Hou Y. Platelets are versatile cells: New discoveries in hemostasis, thrombosis, immune responses, tumor metastasis and beyond. Crit Rev Clin Lab Sci. (2016) 53:409–30. 10.1080/10408363.2016.1200008 27282765

[29] Gong P Liu Y Gong Y Chen G Zhang X Wang S. The association of neutrophil to lymphocyte ratio, platelet to lymphocyte ratio, and lymphocyte to monocyte ratio with post-thrombolysis early neurological outcomes in patients with acute ischemic stroke. J Neuroinflammation. (2021) 18:51. 10.1186/s12974-021-02090-6 33610168 PMC7896410

[30] Massiot N Lareyre F Voury-Pons A Pelletier Y Chikande J Carboni J. High neutrophil to lymphocyte ratio and platelet to lymphocyte ratio are associated with symptomatic internal carotid artery stenosis. J Stroke CerebrovascDis. (2019) 28:76–83. 10.1016/j.jstrokecerebrovasdis.2018.09.001 30268367

[31] Huang G Chen H Wang Q Hong X Hu P Xiao M. High platelet-to-lymphocyte ratio are associated with post-stroke depression. J Affect Disord. (2019) 246:105–11. 10.1016/j.jad.2018.12.012 30578944

[32] Colicchia M Perrella G Gant P Rayes J. Novel mechanisms of thrombo-inflammation during infection: spotlight on neutrophil extracellular trap-mediated platelet activation. Res Pract Thromb Haemost. (2023) 7:100116. 10.1016/j.rpth.2023.100116 37063765 PMC10099327

[33] Acharya P Jakobleff WA Forest SJ Chinnadurai T Mellas N Patel SR. Fibrinogen albumin ratio and ischemic stroke during venoarterial extracorporeal membrane oxygenation. ASAIO J. (2020) 66:277–82. 10.1097/MAT.0000000000000992 30973402 PMC7666805

[34] Wang A Zhang Y Xia G Tian X Zuo Y Chen P. Association of serum albumin to globulin ratio with outcomes in acute ischemic stroke. CNS Neurosci Ther. (2023) 29:1357–67. 10.1111/cns.14108 36794538 PMC10068453

[35] Pascoe MC Skoog I Blomstrand C Linden T. Albumin and depression in elderly stroke survivors: an observational cohort study. Psychiatry Res. (2015) 230:658–63. 10.1016/j.psychres.2015.10.023 26520562

[36] Kitagawa K Hosomi N Nagai Y Kagimura T Ohtsuki T Maruyama H. Cumulative effects of LDL cholesterol and CRP levels on recurrent stroke and TIA. J Atheroscler Thromb. (2019) 26:432–41. 10.5551/jat.45989 30318492 PMC6514170

[37] Cui C Li Q Li C Zhao S Li Y. Statin pretreatment combined with intravenous thrombolysis for ischemic stroke patients: a meta-analysis. J Clin Neurosci. (2022) 98:142–8. 10.1016/j.jocn.2022.02.012 35180504

