# Supplementary material for: The mediation of systemic inflammation on insulin resistance and poor prognosis in non-diabetic ischemic stroke patients treated with intravenous thrombolysis
Source: Front Neurol. 2025 Jul 8;16:1580862. doi: 10.3389/fneur.2025.1580862 (PMC12282244; doi:10.3389/fneur.2025.1580862)

**Supplementary materials**

| **Table I. Demographics and Clinical Characteristics of the Study Population Based on the Propensity Score Matching Dataset [n (%), mean ± SD]** | | | | |
| --- | --- | --- | --- | --- |
| **Characteristics** | **Good prognosis**  **group（n=131）** | **Poor prognosis**  **group（n=74）** | ***P* value** | **Std. Mean Diff.** |
| **Demographic data** |  |  |  |  |
| Sex men | 78 (59.5%) | 47 (63.5%) | 0.681 | -0.083 |
| Age(year) | 68.30 ±11.05 | 68.14 ±12.17 | 0.922 | -0.087 |
| **Stroke risk factors** |  |  |  |  |
| Smoking | 50 (38.2%) | 31 (41.9%) | 0.708 | 0.068 |
| Drinking history | 45 (34.4%) | 24 (32.4%) | 0.9 | -0.014 |
| Hypertension | 101 (77.1%) | 58 (78.4%) | 0.971 | 0.032 |
| CHD | 19 (14.5%) | 12 (16.2%) | 0.9 | 0.036 |
| Atrial fibrillation | 17 (13.0%) | 11 (14.9%) | 0.868 | 0 |
| Hyperlipemia | 29 (22.1%) | 14 (18.9%) | 0.715 | -0.065 |
| History of stroke | 1 ( 0.8%) | 2 ( 2.7%) | 0.614 | 0.088 |
| **Clinical data** |  |  |  |  |
| BMI(kg/m2) | 24.10± 3.43 | 24.43 ±3.32 | 0.503 | 0.113 |
| SBP(mmHg) | 145.13± 16.11 | 148.09± 26.67 | 0.322 | 0.095 |
| DBP(mmHg) | 82.43±11.94 | 84.64 ±16.17 | 0.266 | 0.119 |
| TOAST classification |  |  | 0.064 | 0 |
| LAA | 44 (33.6) | 31 (41.9) |  |  |
| SAO | 78 (59.5) | 32 (43.2) |  |  |
| CE | 9 ( 6.9) | 10 (13.5) |  |  |
| SOE+SUE | 0 ( 0.0) | 1 ( 1.4) |  |  |
| DNT(min) | 38.51 ±5.34) | 39.12 ±6.98) | 0.484 | 0.068 |
| Admission NIHSS score | 5.78 ±4.11 | 6.51 ±4.20 | 0.224 | -0.017 |

Abbreviations: BMI, body mass index; SBP, systolic blood pressure; DBP, diastolic blood pressure; CHD, coronary heart disease; LAA, large artery atherosclerosis; SAO, small artery occlusion; CE, cardioembolism; SOE, stroke of other determined etiology; SUE, stroke of undetermined etiology; DNT, door-to-needle time;

| **Table II. Univariate and Multivariate Logistic Regression Analysis of the study based on propensity score matching data set** | | | | | | |
| --- | --- | --- | --- | --- | --- | --- |
| **Exposure** | **Model (1)** | |  | **Model (2)** | |  |
|  | **OR (95% CI)** | ***P*‐value** |  | **OR (95% CI)** | ***P*‐value** |  |
| **TyG** |  |  |  |  |  |  |
| continuous | 2.85 (1.91–4.25) | <0.001 |  | 2.85 (1.91–4.25) | <0.001 |  |
| categorical |  |  |  |  |  |  |
| TyG＜7.378 | – |  |  | – |  |  |
| TyG＞7.378 | 3.12 (2.01–4.83) | <0.001 |  | 2.88 (1.84–4.50) | <0.001 |  |
| **NLR** |  |  |  |  |  |  |
| continuous | 1.20 (1.09–1.32) | <0.001 |  | 1.20 (1.09–1.32) | <0.001 |  |
| categorical |  |  |  |  |  |  |
| NLR＜3.563 | – |  |  | – |  |  |
| NLR＞3.563 | 1.73 (1.28–2.33) | <0.001 |  | 1.66 (1.21–2.28) | <0.001 |  |
| **PLR** |  |  |  |  |  |  |
| continuous | 0.78 (0.61–0.99) | 0.045 |  | 0.81 (0.63–1.05) | 0.065 |  |
| categorical |  |  |  |  |  |  |
| PLR＜161.94 | – |  |  | – |  |  |
| PLR＞161.94 | 0.78 (0.61–0.99) | 0.045 |  | 0.81 (0.63–1.05) | 0.065 |  |
| **CRP** |  |  |  |  |  |  |
| continuous | 0.96 (0.92–1.00) | 0.061 |  | 0.97 (0.93–1.02) | 0.13 |  |
| categorical |  |  |  |  |  |  |
| CRP＜1.035 | – |  |  | – |  |  |
| CRP＞1.035 | 0.79 (0.62–1.01) | 0.058 |  | 0.82 (0.64–1.06) | 0.074 |  |
| **IPI** |  |  |  |  |  |  |
| continuous | 1.36 (1.05–1.76) | 0.017 |  | 1.34 (1.03–1.75) | 0.024 |  |
| categorical |  |  |  |  |  |  |
| IPI＜0.254 | – |  |  | – |  |  |
| IPI＞0.254 | 1.92 (1.12–3.28) | 0.018 |  | 1.85 (1.07–3.18) | 0.026 |  |

Model (1): no adjustment for covariates.

Model (2): adjusted for BMI, DBP and SBP.

**Table III. Mediation Analysis of the Effect of TyG on Poor prognosis by Inflammatory Markers (After PSM)**

| **Mediator** | **Total Effect** | | **Indirect Effect** | | **Direct Effect** | | **Proportion Mediated** |
| --- | --- | --- | --- | --- | --- | --- | --- |
|  | **Coefficient**  **(95% CI)** | ***P*‐value** | **Coefficient**  **(95% CI)** | ***P*‐value** | **Coefficient**  **(95% CI)** | ***P*‐value** |  |
| NLR | 0.334 (0.227–0.460) | <0.001 | 0.064 (0.030–0.112) | 0.001 | 0.279 (0.182–0.410) | <0.001 | 19.20% |
| IPI | 0.328 (0.222–0.452) | <0.001 | 0.052 (0.020–0.098) | 0.005 | 0.287 (0.186–0.416) | <0.001 | 15.90% |

**Figure S1. Flowchart of patient selection.**


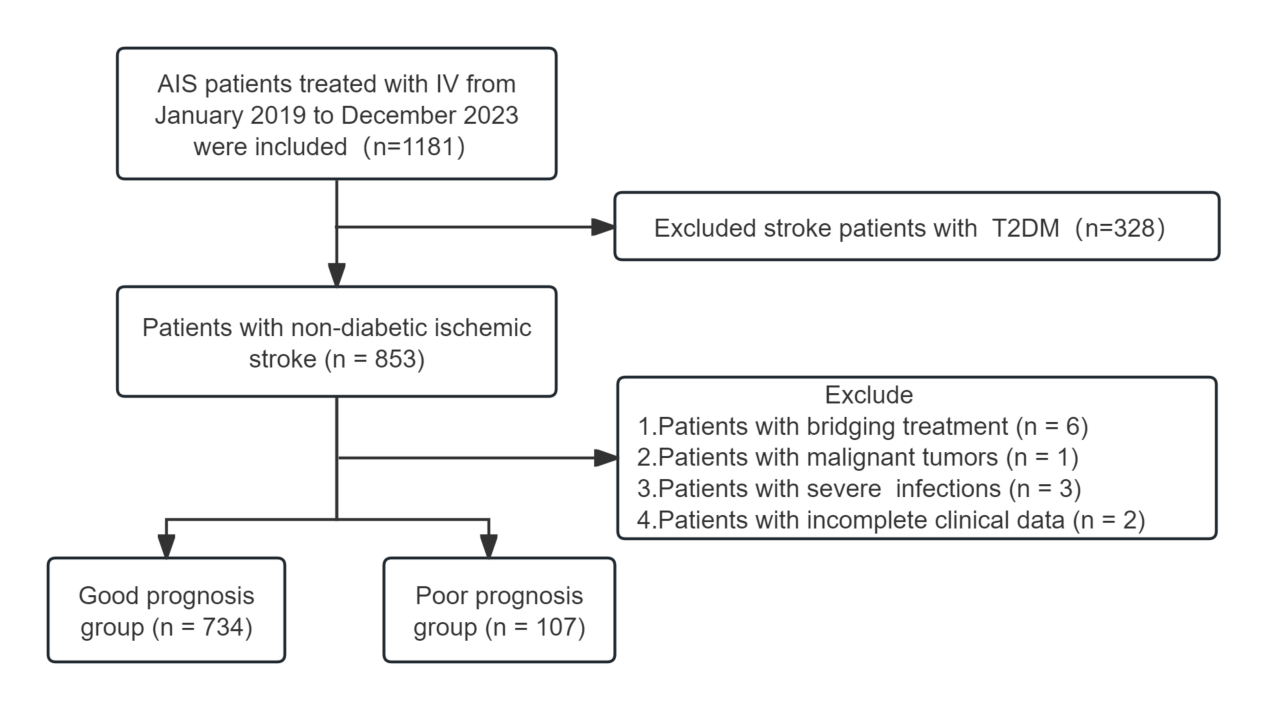


**Figure S2. Subgroup analysis of the association between TyG index and poor prognosis in nondiabetic patients treated with IVT after PSM**
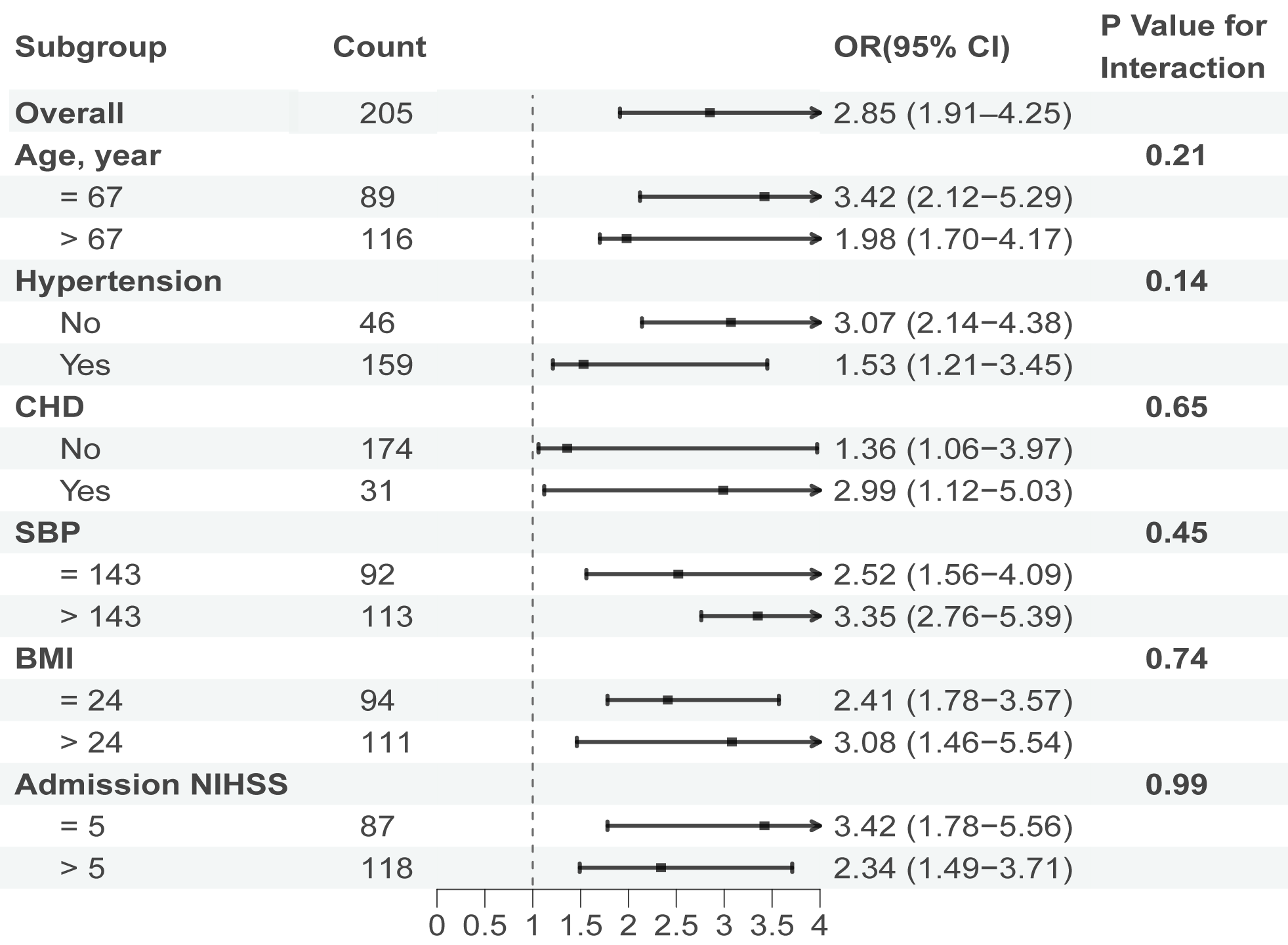

Supplement: Supplementary file 1 [file Table_1.docx]
